# Low *DLG2* gene expression, a link between 11q-deleted and *MYCN-*amplified neuroblastoma, causes forced cell cycle progression, and predicts poor patient survival

**DOI:** 10.1186/s12964-020-00553-6

**Published:** 2020-04-20

**Authors:** Simon Keane, Sophie Améen, Angelica Lindlöf, Katarina Ejeskär

**Affiliations:** 1 Translational Medicine, School of Health Sciences, University of Skövde, PO Box 408, SE-54128 Skövde, Sweden; 2 Translational Bioinformatics, School of Biosciences, University of Skövde, Skövde, Sweden

**Keywords:** Neuroblastoma, 11q, MAGUK, DLG2, MYCN

## Abstract

**Background:**

Neuroblastoma (NB) is a childhood neural crest tumor. There are two groups of aggressive NBs, one with *MYCN* amplification, and another with 11q chromosomal deletion; these chromosomal aberrations are generally mutually exclusive. The *DLG2* gene resides in the 11q-deleted region, thus makes it an interesting NB candidate tumor suppressor gene.

**Methods:**

We evaluated the association of *DLG2* gene expression in NB with patient outcomes, stage and *MYCN* status, using online microarray data combining independent NB patient data sets. Functional studies were also conducted using NB cell models and the fruit fly.

**Results:**

Using the array data we concluded that higher *DLG2* expression was positively correlated to patient survival. We could also see that expression of *DLG2* was inversely correlated with *MYCN* status and tumor stage. Cell proliferation was lowered in both 11q-normal and 11q-deleted NB cells after *DLG2* over expression, and increased in 11q-normal NB cells after *DLG2* silencing. Higher level of *DLG2* increased the percentage of cells in the G2/M phase and decreased the percentage of cells in the G1 phase. We detected increased protein levels of Cyclin A and Cyclin B in fruit fly models either over expressing dMyc or with RNAi-silenced *dmDLG*, indicating that both events resulted in enhanced cell cycling. Induced *MYCN* expression in NB cells lowered *DLG2* gene expression, which was confirmed in the fly; when dMyc was over expressed, the dmDLG protein level was lowered, indicating a link between Myc over expression and low dmDLG level.

**Conclusion:**

We conclude that low *DLG2* expression level forces cell cycle progression, and that it predicts poor NB patient survival. The low *DLG2* expression level could be caused by either *MYCN*-amplification or 11q-deletion.

**Graphical abstract:**

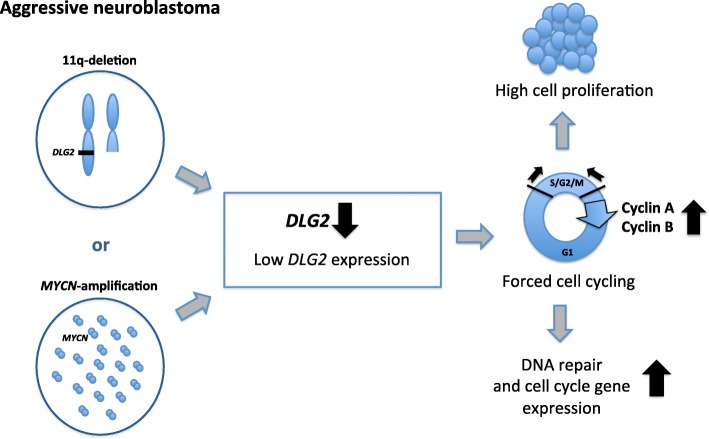

**Video Abstract**

## Background

Neuroblastoma (NB) is a tumor arising from the transient embryonic neural crest, later presenting in the sympathetic division of the autonomous nervous system [1]. It is one of the most common forms of extra cranial solid tumors found in children aged under two [2]. The clinical diagnosis of NB is difficult in part due to the age of the patient and the vague appearance of the symptoms [3]. NB is post surgically staged according to the International Neuroblastoma Staging System (INSS). INSS stages 1 and 2 are complete or partially resected localized tumors; stage 3 denotes the larger localized tumors that cross the midline. Stage 4 tumors have dissemination of the tumor to lymph nodes or bone marrow. Stage 4S are special cases, where the patient is younger than 1 year old, having a one sided tumor with metastasis to the liver or skin but limited bone marrow involvement [4]. The survival prognosis of the 4S stage patients is lower than the stage 2 tumor group but better than the stage 3 tumor group [5].

NB can also be defined by risk group, low and intermediate NB have a good prospect for treatment, whereas, the high risk tumors are more difficult to treat [6] and which results in a higher mortality. High-risk NB tumors have specific genetic characteristics and are highly aggressive. Two of the most prevalent forms of high risk NB are; amplification of the *MYCN* proto-oncogene [7, 8] and unbalanced 11q-deleted loss of heterozygosity (LOH) tumors [2, 9], which account for approximately 20 and 30%, respectively, of all cases. Patients with 11q LOH are always heterozygous for the deletion and generally lack *MYCN* amplification [10]. However, in the rare cases when patients do have both *MYCN* amplification and 11q LOH, the deletion point is found to be located more terminally than other 11q LOH tumors. These two common aggressive NB forms are therefore usually considered to be mutually exclusive [11]. The mechanism of the mutual exclusivity remains to be determined, as the definitive identification of a single tumor suppressor gene within 11q remains elusive. Consequently, it is accepted that the tumor suppressor gene disrupted by 11q LOH must satisfy a set of criteria based on the known clinical and genetic alterations observed, something that previous attempts at tumor suppressor characterization on 11q have also considered. The first characteristic is the inverse relation of 11q and *MYCN* amplification; with 11q deletion substituting the function of *MYCN* amplification or by *MYCN* amplification acting to disrupt the 11q tumor suppressor gene function [11]. Secondly, the tumor suppressor should maintain genome integrity and prevent the increased number of chromosomal breaks that are observed in the 11q LOH tumors [2]. Finally, there should also be a suitable two hit mechanism to account for the deletion always appearing as heterozygous only deletion [12, 13]. To date a second hit has not been found in the proposed 11q tumor suppressor genes, including; *CADM1* (11q23.3) [14], *ATM* (11q22.3) [15] and *H2AFX* (11q23.3) [11].

Since the identification of 11q-deleted NB, debate has raged over the smallest region of overlap (SRO) of the deletion. Initially, the SRO was identified at 11q23 [16], subsequent identification showed that the deletion extended to 11q14 [10]. Now it has been suggested that there are three separate SROs on 11q; the first, an amplification from 11q13.2 to 11q13.4, the second, a deletion spanning from 11q14.1 to 11q22.2 and finally a deletion spanning 11q23.1 to 11q23.3 for the rare tumors with both 11q deletion and *MYCN* amplification [17]. Located within the 11q14.1 SRO, and always deleted in the 11q-deleted NBs without *MYCN* amplification, is one of the Discs Large homolog (DLG) family members, *DLG2*. The DLG family has important functions governing polarity, cellular structure and growth behavior [18–20]. These functions are thought to be achieved by protein trafficking to the cellular surface of epithelial cells as well as the organization and stabilization of supra-molecular adhesion and signaling complexes through heterodimeric formation [21]. Orthologous to the human *DLG2* gene is the *Drosophila melanogaster* DLG gene *(dmDLG)*. *Drosophila melanogaster* only contain a single orthologue to the human DLG family. Loss or mutation of *dmDLG* is known to result in spontaneous neoplasms [22]. *dmDLG* was identified early as a tumor suppressor gene along with complex partners scribble (*Scrib*) and lethal giant larvae (*LGL*) [23]. Knockdown of *dmDLG* has been implicated in early and abnormal exit from the cell cycle and can result in binucleate cells [24]. *dmDLG* has also been shown to be important in *Drosophila* spindle alignment in asymmetric neuroblast division [25]. Recently, abnormally low *DLG2* expression in the human cancers osteosarcoma [26] and ovarian cancer [27] has been identified. GWAS studies have also shown that an intronic SNP (rs790356) within *DLG2* correlates to nephroblastoma, a common childhood renal tumor [28]. Therefore, *DLG2* is a good candidate to be the 11q tumor suppressor gene in NB.

In this study we have considered *DLG2* to be a functional and positional tumor suppressor gene in 11q-deleted NB. We have investigated this using clinical pathology data and gene expression in both primary NBs and in NB cell lines. We have confirmed this data by *DLG2* over expression and silencing experiments in NB cell lines and in the fruit fly.

## Methods

### Gene expression analysis

Data for analyses and comparison of *DLG2* expression between the different NB patient subgroups was imported from the R2 platform (http://r2.amc.nl). The independent NB primary datasets; 1): SEQC GSE49710 (microarray), 2): Versteeg GSE16476 (microarray), 3): Maris GSE3960 (microarray) and 4): Westermann GSE73517 (microarray). Neuroblastoma methylation datasets; 1): Westermann GSE73515 (Methylation array) and 2): Fisher GSE120650 (Methylation array). The neuroblastoma cell datasets; Maris; 1): GSE89413 (Cell Line, RNA-Seq), 2): Versteeg GSE28019 (Cell Line, microarray), 3), Versteeg GSE16478 (Cell Line, microarray), and human embryogenesis dataset 1): Yi GSE15744 (microarray). The microarray data was downloaded as the centered log2 fold change. Methylation data was downloaded as raw values.

### Cell lines and tissue culture

Human NB cell line SKNAS and NB69 were obtained from ATCC Cell Line Collection. The cell lines were maintained in RPMI 1640 supplemented with 10% FBS, 1% L-Glutamine, 1% HEPES solution and 1% sodium pyruvate. Cells were maintained at 37 °C with 5% CO_2_.

### Plasmids, siRNAs and transfections

*DLG2* (NM_001364) expression plasmids on a backbone of pCMV6-AC-GFP (catalogue # PS100010) vector were purchased from Origene Technologies. siRNA targeting *DLG2* (s4122) or Silencer™ Select Negative control No. 1 siRNA (4390843) was purchased from Ambion (Thermo Fischer Scientific). SKNAS and NB69 cells were grown to 80% confluence and subsequently transfected with; *DLG2* plasmid, empty vector “mock” (pCMV6-AC-GFP), si-*DLG2* or scrambled control “mock”. 100 ng of plasmid DNA or 10 pmol siRNA was complexed with 0.3 μl of Lipofectamine 2000 according to the Lipofectamine 2000 reagent forward transfection protocol (Invitrogen; Thermo Fisher Scientific).

### Cell viability, proliferation and cell cycle analysis

100 μl cell suspension of SKNAS and NB69 (1 × 10^4^ cells/well) was seeded in 96-well culture plates (Corning Incorporated). After culturing to 80% confluence the supernatant was removed and transfection media was added to the cells. 48 h post transfection, cells were counted using a 60 μm sensor for the Scepter handheld cell counter (Millipore) as per the manufactures instructions [29]. Cell proliferation was measured using the MTS/MPS Cell Titer 96® One solution Reagent (Promega) and detecting the color variation (FLUOstar Omega, BMG Labtech) as per the manufacturer’s recommendations. The absorbance values were normalized to the mock transfection and expressed as a percentage. All experiments were repeated three times.

Cell cycle analysis was performed using the Cell-clock cell cycle assay (Biocolor). Images were subsequently analyzed using Image J image analysis as per the manufacturer’s instructions. The data presented is the average of three biological replicates. Each experiment series was repeated in triplicate.

### Fly strains and crosses

Commercially available control white (w1118) flies, UAS-dMYC and UAS-RNAi-dlg1 flies were crossed with da-GAL4 driver strain to ubiquitously force or silence gene expression, all strains were obtained from the Bloomington stock center (Bloomington). Twenty female da-GAL4 flies were crossed with 10 male UAS-transgenic flies or control flies and the progeny incubated at 25 °C on standard fly media. Five of the progeny were harvested after 72 h during the third instar larvae phase. Each cross was controlled using the inverse cross using 20 UAS-transgenic female flies crossed with 10 da-GAL4 male flies.

### Protein analysis by Western blot

Protein was extracted from the transfected cells in 96 well plates (1 × 10^4^ cells/well), by aspirating the media and incubating on ice for 5 min then adding ice cold RIPA buffer (Thermo-fisher Scientific, 89900). For fly protein extractions 5 larvae were homogenized in RIPA buffer, followed by centrifugation. Western blot analysis was performed using a Mini-PROTEAN® TGX™ 8–20% gradient gel (Bio-Rad), protein was blotted onto LF-PVDF membrane (8 min, 25 V and 2.5A) using a Trans-Blot® Turbo™ Transfer System (Bio-Rad). Blots were subsequently blocked for 1 h in 5% milk in TBST buffer (0.1% Tween-20 and 150 mM NaCl in 10 mM Tris–HCL, pH 7.4) as per the manufacturer’s recommendations. Blots were probed overnight at 4 °C with antibodies diluted in PBST (0.1% Tween-20 in PBS). Primary antibodies; dmDLG (4F3, anti-discs large, Goodman, C.), Cyclin A (A12, Lehner, C.F.), Cyclin B (F2F4, O’Farrell, P.H.) and α-tubulin (12G10, Frankel, J. / Nelsen, E.M.) were obtained from the Developmental Studies Hybridoma Bank, created by the NICHD of the NIH and maintained at The University of Iowa, Department of Biology, Iowa City, IA 52242. All primary antibodies were diluted to 0.5 μg/ml in PBST 0.1%. The secondary antibody used for detection was Starbright goat anti-mouse 1:5000 (12004159, Bio-Rad). All wash stages were 3 × 10 minutes in TBST 0.1%. Secondary antibodies were incubated for 1 h at room temperature. Image detection was performed on ChemiDoc MP (Bio-Rad).

### Quantitative PCR analysis

RNA from NB cell lines was extracted with RNeasy Kit® (Qiagen) according to manufacturer’s protocol. RNA was quantified by NanoDrop (NanoDrop Technologies) and 2 μg of RNA was reverse transcribed into double stranded cDNA on a T-professional Basic Gradient thermal cycler (Biometra) using the High Capacity cDNA Reverse Transcription kit (Applied Biosystems). cDNA corresponding to 20 ng of RNA was used for each qPCR reaction. qPCR was performed on a Pikoreal qPCR System (Thermo Fischer Scientific) in triplicate for TaqMan target transcripts; *DLG2* (Hs00265843_m1) and *dmDLG* (Dm01799281_g1) using TaqMan™ Gene Expression Master Mix ( Applied Biosystems). Quantitative gene expression data were normalized to the expression levels of the human reference genes *GAPDH* (Hs02758991_m1), *GUSB* (Hs99999904_m1) and fly reference gene *Rpl32* (Dm02151827_g1).

### Gene set enrichment analysis

To identify the pathways to which *DLG2* expression is correlated with, the previously described independent datasets; 1, 2 and 3 were selected from the R2 platform (http://r2.amc.nl). A gene expression list was derived based on the correlation to *DLG2* expression, normalized to z-score. To further investigate enriched pathways, the non-NB developmental dataset 10 was selected for additional comparison. The top 10 enriched KEGG pathways were subsequently shown for each dataset using the R2 platform. Data was corrected for multiple comparisons using the false discovery rate (FDR). Concordance between the datasets was shown using Venn diagrams produced with the geneVenn tool (genevenn.sourceforge.net).

### Statistical analysis

All data is presented as Tukeys box and whisker plots showing IQR, line at the median, + at the mean with whiskers ±1.5-fold of interquartile range from at least 3 independent experiments. For all multi-group analyses, differences were determined by one way ANOVA test followed by Holm-Sidak’s multiple comparison test. For comparisons between two groups a Mann-Whitney U test was used. For determination of overall and event free survivals the Kaplan Meier estimator was used with differences between groups determined using a two sided log rank test. ^*^p < 0.05, ^**^p < 0.01, ^***^p < 0.001. All analyses were conducted using GraphPad Prism version 8.0.1 for Windows, (GraphPad Software, www.graphpad.com).

## Results

### *DLG2* expression is low in high INSS stage NB and correlates to survival

**Fig. 1:**
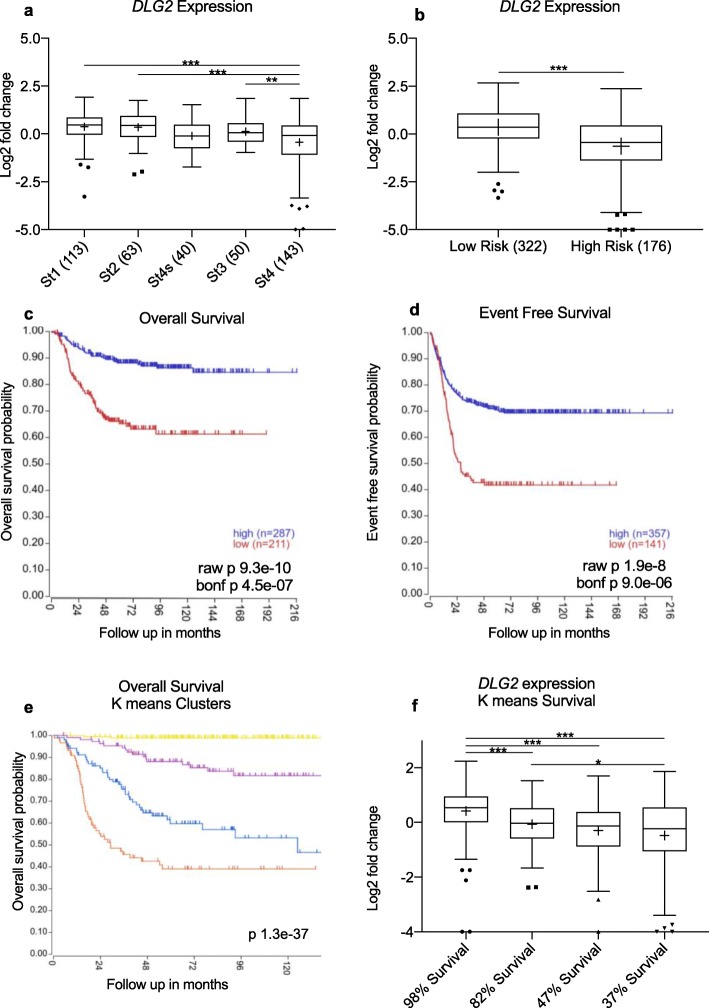
*DLG2* expression in NB correlates with survival and stage. *DLG2* gene expression of NB primary dataset 1 (*GSE49710*) stratified by **a** INSS stage and **b** by risk category in primary NB. **c, d** and **e** are Kaplan-Meier plots showing the overall survival probability, the event free survival and the overall survival of the k means 4 groups clustering. **f***DLG2* expression stratified by K means clustering. The expression data are presented as median centered log2 fold change and plotted as Tukeys box and whisker plots showing IQR, line at the median, + at the mean with whiskers ±1.5-fold of interquartile range. Data outside the whiskers are shown as outliers. ^*^p < 0.05, ^**^p < 0.01, ^***^p < 0.001. Kaplan-Meier plots are determined using the product limit estimator followed by Bonferroni correction

We evaluated the association of *DLG2* expression with INSS stage as well as patient outcomes, using the NB primary dataset 1 (GSE49710) obtained from the R2 Genomics Analysis and Visualization Platform (http://r2.amc.nl). When comparing *DLG2* expression level between INSS stages, stage 4 tumors showed significantly lower *DLG2* expression when compared to stage 1 tumors (log2 FC = 0.89, *p* < 0.001), stage 2 tumors (log2 FC = 0.83, *p* < 0.001) and stage 3 tumors (log2 FC = 0.55, *p* < 0.005), with the only exception of stage 4 s tumors (Fig. 1a). A similar trend was also observed in the NB primary datasets 2, 3 and 4 (Fig. S1a, b and d respectively), with the stage 4 tumors showing low *DLG2* expression. There was also a significant difference in the expression of *DLG2* in the high risk and low risk patients (log2 FC = 0.80, *p* < 0.001) (Fig. 1b). The same trend was also observed in NB primary dataset 4 (Fig. S1f). Overall survival outcomes (Fig. 1c) and event free survival (Fig. 1d) for the patients were determined using Kaplan-Meier analysis using NB primary dataset 1. High expression of *DLG2* was associated with increased probability of both overall and event free survival (Fig. 1c-d, *p* < 0.001).). The same trend was also observed in NB primary dataset 2 (Fig. S2a and S2b). The gene expression data was subsequently clustered into 4 groups using the normalized total array expression and K means clustering method, and the survival of each of the 4 groups was then determined using Kaplan-Meier analysis. One of the groups showed a high survival, with 98% overall survival, while the other groups showed 82%, 47% respectively 37% survival (Fig. 1e). The groups were subsequently named after their overall survival and *DLG2* expression was determined for each group. The group with 98% survival also had the highest *DLG2* expression compared to 82% survival (log2 FC = 0.49, *p* < 0.001), 47% survival (log2 FC = 0.71, *p* < 0.001) and 37% survival (log2 FC = 0.90, *p* < 0.001), there was also a significantly lower expression of *DLG2* in the 37% survival group when compared to the 82% survival group (log2 FC = 0.41, *p* < 0.05) (Fig. 1f). The similar K means clustering results was also observed in NB primary dataset 2 (Fig. S2c and S2d).

### Gene set enrichment analysis shows *DLG2* inversely correlates to cell cycle genes

**Fig. 2:**
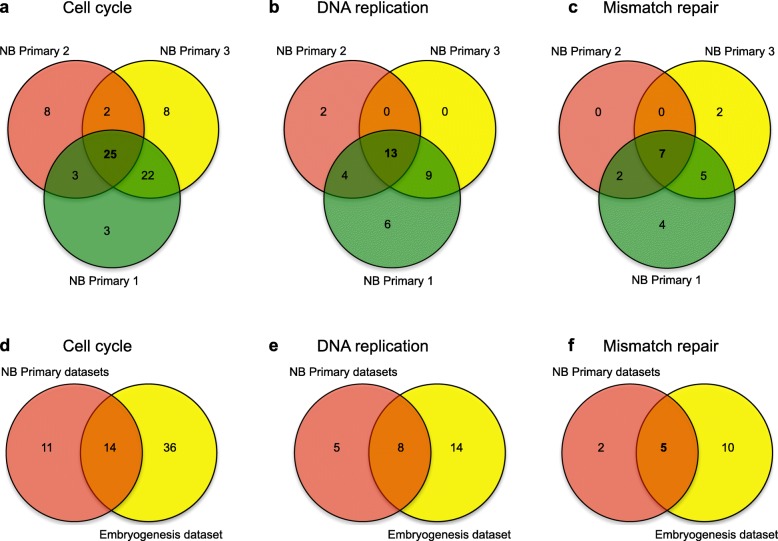
Coalescence analysis of cell cycle, DNA repair and mismatch repair pathway genes. Genes correlated with *DLG2* expression and involved in enriched pathways were analyzed for the concordance between NB primary dataset 1, 2 and 3; regarding **a** cell cycle genes, **b** DNA replication genes and **c** mismatch repair genes. Identified concordant genes from previous step were analyzed for concordance with human embryogenesis dataset 1; regarding **d** cell cycle genes, **e** DNA replication genes and **f** mismatch repair genes. Numbering marks the number of genes in common for the datasets

Gene lists were created for the genes correlating to *DLG2* expression in NB primary datasets 1 (*GSE49710)*, 2 (GSE16476) and 3 (GSE3960), and an embryogenesis dataset 1 (GSE15744). The NB primary dataset 4 was not analysed due to the customized probe layout, and the cell line datasets were also not included. Gene set enrichment analysis of the genes correlating to *DLG2* in the selected data sets showed that the pathways; cell cycle (*p* < 0.0001), DNA replication (*p* < 0.0001), Fanconi anemia (*p* < 0.0001) and mismatch repair (*p* < 0.0001) were enriched in the three analyzed NB primary datasets (Table 1, 2 and 3). When these four identified pathways were compared to the enriched pathways in the embryogenesis dataset (Table 4), the Fanconi anemia pathway did not appear. The proportion of cell cycle genes in NB primary datasets 1, 2 and 3 were 71.8%; 31.4% and 47.7%, respectively; (*p* < 0.0001), DNA replication genes 88.9%; 52.8% and 78.1%, respectively (*p* < 0.0001), mismatch repair genes 78.3%; 40.9% and 70.0%, respectively (*p* < 0.0001) and Fanconi anemia pathway genes 69.8%; 42.2% and 60.0%, respectively (*p* < 0.0001) (Table 1, 2 and 3). The embryogenesis dataset showed 41.7% cell cycle genes (*p* < 0.001), 61.1% DNA replication genes (*p* < 0.0001) and 63.6% mismatch repair genes (*p* < 0.0001) (Table 4). The genes involved in the enriched pathways showed an overriding negative correlation to *DLG2* expression*.* Concordance between the enriched pathways for the NB primary datasets 1, 2 and 3 was first determined using the intersection of these three datasets and subsequently compared to the embryogenesis dataset. The intersect of the cell cycle in the NB datasets included 25/71 genes (Fig. 2a), 13/35 genes for DNA replication (Fig. 2b), and 7/20 mismatch repair genes (Fig. 2c), with the concordant genes listed in the supplementary data (Table S1). When compared to the embryogenesis dataset the intersection included 14/61 genes for cell cycle (Fig. 2d), 8/27 genes for DNA replication (Fig. 2e) and 5/17 genes for mismatch repair (Fig. 2f); these concordant genes are found in Table 5.

**Table 1:** GSEA of genes correlated to *DLG2* expression in NB primary dataset 1 (GSE49710)

Enriched KEGG Pathway	# correlated genes	# pathway genes in total	Percentage	Corrected ***p***-value
Ribosome	124	133	93.2%	5.2e-25
Oxidative phosphorylation	93	120	77.5%	2.0e-10
Alzheimer s disease	115	162	71.0%	9.7e-09
Cell cycle	89	124	71.8%	2.1e-07
Huntington’s disease	126	187	67.4%	2.3e-07
DNA replication	32	36	88.9%	1.2e-06
Sphingolipid signaling pathway	84	120	70.0%	2.4e-06
Spliceosome	89	131	67.9%	8.2e-06
Fanconi anemia pathway	37	53	69.8%	1.9e-03
Mismatch repair	18	23	78.3%	4.3e-03

**Table 2:** GSEA of genes correlated to *DLG2* expression in NB primary dataset 2 (GSE16476)

Enriched KEGG Pathway	# correlated genes	# pathway genes in total	Percentage	Corrected ***p***-value
DNA replication	19	36	52.8%	2.1e-11
Ribosome biogenesis in eukaryotes	27	64	42.2%	8.4e-11
RNA transport	44	139	31.7%	2.1e-09
Cell cycle	38	121	31.4%	3.6e-08
Fanconi anemia pathway	19	45	42.2%	5.0e-08
Spliceosome	37	119	31.1%	8.0e-08
Pyrimidine metabolism	26	94	27.7%	1.4e-04
Homologous recombination	11	29	37.9%	2.1e-04
Mismatch repair	9	22	40.9%	2.8e-04
Non homologous end joining	5	11	45.5%	2.7e-03

**Table 3:** GSEA of genes correlated to *DLG2* expression in NB primary dataset 3 (GSE3960)

KEGG Pathway	# correlated genes	# pathway genes in total	Percentage	Corrected ***p***-value
Ribosome	57	70	81.4%	4.50e-20
DNA replication	25	32	78.1%	6.70e-09
Oocyte meiosis	42	80	52.5%	2.60e-05
Pyrimidine metabolism	34	63	54.0%	6.80e-05
Cell cycle	53	111	47.7%	1.10e-04
Mismatch repair	14	20	70.0%	1.50e-04
Spliceosome	45	95	47.4%	4.70e-04
One carbon pool by folate	9	12	75.0%	9.10e-04
Fanconi anemia pathway	15	25	60.0%	1.60e-03
Folate biosynthesis	8	11	72.7%	2.60e-03

**Table 4:** GSEA of genes correlated to *DLG2* expression in the human embryogenesis dataset (GSE15744)

KEGG Pathway	# correlated genes	# pathway genes in total	Percentage	Corrected ***p***-value
One carbon pool by folate	13	17	76.5%	3.3e-05
Spliceosome	56	118	47.5%	4.5e-05
DNA replication	22	36	61.1%	5.4e-05
ECM receptor interaction	36	70	51.4%	1.1e-04
Mismatch repair	14	22	63.6%	6.4e-04
Protein digestion and absorption	33	67	49.3%	6.9e-04
Glycolysis Gluconeogenesis	27	57	47.4%	4.8e-03
Salivary secretion	25	52	48.1%	5.0e-03
Pyruvate metabolism	18	35	51.4%	6.3e-03
Cell cycle	50	120	41.7%	6.3e-03

**Table 5:** Cell cycle, DNA replication and mismatch repair genes negatively correlated to *DLG2* expression, common to NB primary datasets 1–3 (GSE49710, GSE16476 and GSE89413) and the human embryogenesis dataset (GSE15744)

Gene symbol	Gene function	Cellular process
*BUB1, BUB1B*	Mitotic checkpoint serine/threonine kinase	Mitotic checkpoint
*CCNA2*	Cyclin A2, G1/S to G2/M transition	Cell cycle regulation
*CCNB1*	Cyclin B1, G2/M transition	Cell cycle regulation
*CDC20*	Cell division, anaphase regulation	Chromosome separation
*CDK4*	Cyclin dependent kinase, G1/S phase	Cell cycle regulation
*DBF4*	E2F mediated regulation of DNA replication	DNA replication
*ESPL1*	Sister chromatid cohesion and separation	Chromosome separation
*EXO1*	Exonuclease 1	DNA repair
*LIG1*	DNA ligase, DNA replication and repair	DNA replication
*MCM2, MCM3, MCM5, MCM7*	Initiation of genome replication	DNA replication
*MSH6*	Mismatch recognition	DNA repair
*POLD2*	DNA polymerase delta	DNA replication
*PRKDC*	DNA-dependent protein kinase	DNA repair
*POLA1, POLA2*	DNA polymerase alpha	DNA replication
*RFC3, RFC4*	Replication factor, DNA elongation	DNA replication
*TP53*	Tumor suppressor, DNA binding	Cell cycle regulation

#### *MYCN* amplified tumors have low *DLG2* expression

**Fig. 3:**
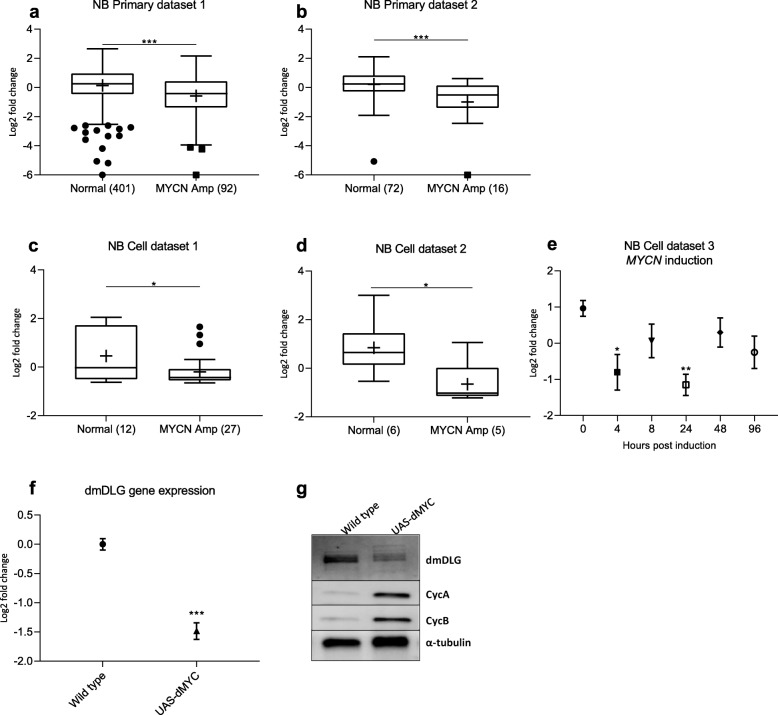
*DLG2* expression in *MYCN* amplified NB. *DLG2* expression level and *MYCN* status in primary NB **a** NB primary dataset 1 (*GSE49710*) and **b** NB primary dataset 2 (*GSE16476*); and in NB cell line datasets **c** NB cell dataset 1 (*GSE89413*) and **d** NB cell dataset 2 (*GSE28019*), bone marrow derived. **e** The effect of *MYCN* induction by doxycycline on *DLG2* expression in SKNAS cells over time from NB cell dataset 3 (*GSE16478*). The data (a-d) are presented as median centered log2 fold change and plotted as Tukeys box and whisker plots showing IQR, line at the median, + at the mean with whiskers ±1.5-fold of interquartile range. Data outside the whiskers are shown as outliers. **f***dmDLG* gene expression in wild type and dMYC over expressing (UAS-dMyc) *Drosophila* larvae. **g** immunoblot of wild type and dMYC over expressing (UAS-dMyc) *Drosophila* larvae targeting *dmDLG*, Cyclin A, Cyclin B normalized to α-tubulin. **e** and **f** are expressed as the mean ± SEM. ^*^p < 0.05, ^**^p < 0.01, ^***^p < 0.001

Gene expression of *DLG2* was found to be significantly lower in *MYCN* amplified samples in 4 independent datasets (1–4), including, two patient cohorts and two cell line cohorts (Fig. 3a-d). The NB primary dataset 1 was used to identify the decrease in *DLG2* expression in *MYCN* amplified samples (log2 FC = 0.71, *p* < 0.0001) (Fig. 3a), with NB primary dataset 2 used to confirm the result (log2 FC = 1.21, *p* = 0.0005) (Fig. 3b). The NB cell dataset 1 (GSE89413) with 39 distinct NB cell lines (27 containing *MYCN* amplification) was used to confirm the lower *DLG2* expression in *MYCN* amplified cell lines (log2 FC = 0.65, *p* = 0.0022) (Fig. 3c). In the NB cell dataset 2 (GSE28019) we controlled for variability in the tissue of origin of the cell lines by using only the cell lines derived from metastatic bone marrow, here the *MYCN* amplified cells also showed lower expression (log2 FC = 1.50, *p* = 0.049) (Fig. 3d). We further noted that induction of *MYCN* in the NB cell dataset 3, (GSE16478), resulted in a decrease in the expression of *DLG2* (Fig. 3e). A similar trend was also observed in the NB primary datasets 3 and 4 (Fig. S1c and S1e) with the *MYCN* amplified tumors showing low *DLG2* expression.

To model the effect of *MYCN* amplification on *DLG2* we investigated the effect of forced expression of the orthologous *Drosophila melanogasterdMYC* gene using a UAS-dMYC construct ubiquitously induced by da-Gal4 in a fruit fly model*,* and investigated changes in dmDLG gene expression and protein level in the larvae. Overexpression of *dMYC* resulted in lower gene expression of *dmDLG* (log2 FC = 0.84, *p* = 0.001) (Fig. 3f). We confirmed the decrease in dmDLG also on protein level (Fig. 3g), and furthermore evaluated the effect of *dMYC* over expression on Cyclin A (cycA) and Cyclin B (cycB). Consistent with the previous literature [30], we could determine an increase in the expression of both cycA and cycB (Fig. 3g).

#### 11q deletion correlates to low DLG2 expression

**Fig. 4:**
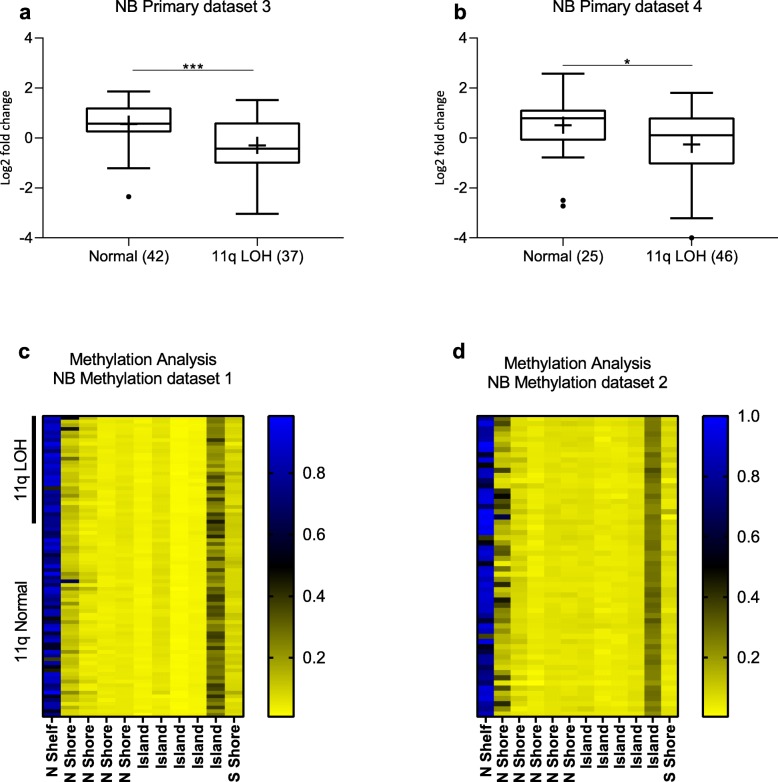
*DLG*2 expression in 11q LOH NB. *DLG2* gene expression in the primary 11q LOH NB **a** NB primary dataset 3 (*GSE3960*) and **b** NB primary dataset 4 (*GSE73517*). Data presented as median centered log2 fold change and plotted as Tukeys box and whisker plots showing IQR, line at the median, + at the mean with whiskers ±1.5-fold of interquartile range. Data outside the whiskers are shown as outliers. Promoter methylation analysis in primary NB **c** NB methylation dataset 1 (GSE73515) and **d** NB methylation dataset 2 (GSE120650), presented as heat maps. ^*^p < 0.05, ^***^p < 0.001

Gene expression of *DLG2* was found to be significantly lower in 11q-deleted NB tumor data when *MYCN* amplification was excluded, in two independent neuroblastoma primary datasets (3 and 4). In primary NB dataset 3 (GSE3960), the 11q-deleted samples showed a significant decrease (log2FC = 0.85, *p* = 0.0004) when compared to the 11q normal tumors (Fig. 4a). NB primary dataset 4 (GSE73517) confirmed that 11q-deleted tumors had lower expression of *DLG2* compared to the 11q normal tumors (log2FC = 0.66, *p* = 0.034) (Fig. 4b). Methylation array data showed that there was very low methylation of the *DLG2* promoter region, no observable difference in the promoter region methylation pattern was observed in 11q-deleted NB compared to 11q normal in NB methylation dataset 1 (GSE73515) (Fig. 4c) and also no *DLG2* promoter methylation in general in NB methylation dataset 2 (GSE120650) (Fig. 4d).

#### *DLG2* silencing or over expression changes the growth behavior of NB cells

**Fig. 5:**
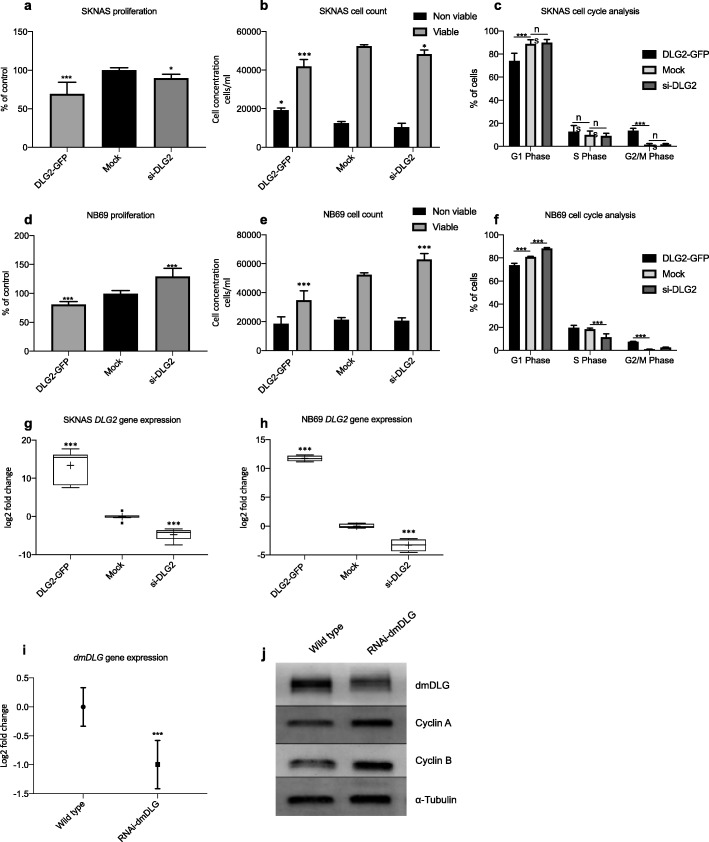
Cell responses after *DLG2* over expression or silencing. Cell responses 48 h post *DLG2* over expression (DLG2-GFP) or silencing (si-DLG2) in SKNAS cells (11q-deleted): **a** proliferation; **b** viable and non-viable cell fraction; **c** cell cycle analysis. Cell responses 48 h post *DLG2* over expression (DLG2-GFP) or silencing (si-DLG2) in NB69 cells (11q-normal): **d** proliferation; **e** viable and non-viable cell fraction; **f** cell cycle analysis. *DLG2* gene expression analysis 48 h post *DLG2* over expression (DLG2-GFP) or silencing (si-DLG2) in **g** SKNAS cells; **h** NB69 cells. **i***dmDLG* gene expression in wild type and *dmDLG* silenced (RNAi-dmDLG) *Drosophila* larvae. **j** immunoblot of wild type and *dmDLG* silenced (RNAi-dmDLG) *Drosophila* larvae targeting dmDLG, Cyclin A, Cyclin B, normalized to α-tubulin. The data in **a-f** shows the mean and SD. **a** and **d** are normalized as a percentage of the mock transfection, *n* = 9. The data shown is the pooled average of 3 experiments. **g** and **h** are expressed as median centered log2 fold change and plotted as Tukeys box and whisker plots showing IQR, line at the median, + at the mean with whiskers ±1.5-fold of interquartile range. ^*^p < 0.05, ^***^p < 0.001, *n* = not significant

Over expression of *DLG2* in 11q-deleted NB cells (SKNAS) resulted in slower proliferation compared to the control (Fig. 5a, *p* < 0.001). We observed a decrease in the number of viable cells (Fig. 5b, *p* < 0.001) and an increase in the non-viable cell fraction (Fig. 5b, *p* < 0.05) in cells with increased *DLG2* expression. *DLG2* silencing in 11q-deleted SKNAS cells resulted in a slight decrease in cell proliferation (Fig. 5a, *p* < 0.05), with no effect in viable/non-viable cell numbers (Fig. 5b). We determined that over expression of *DLG2* in SKNAS resulted in a decrease of the percentage of cells in G1 phase and an increase in the number of cells in G2/M phase (Fig. 5c). There was no difference in the cell cycle state for *DLG2* silenced cells compared to control (Fig. 5c). This was repeated in 11q normal NB cells (NB69), where over expression of *DLG2* resulted in slower proliferation compared to the control (Fig. 5d, *p* < 0.001). There was a significant decrease in total viable cell number (Fig. 5e, *p* < 0.001) with no increase in the nonviable fraction. Knockdown of *DLG2* resulted in an increase in cell proliferation compared to the mock control in 11q normal NB69 cells (Fig. 5d, *p* < 0.001). An increase in the total number of viable cells was also determined (Fig. 5e, *p* < 0.001), with no change in the number of non-viable cells. As there was no alteration in the non-viable fraction after changing the *DLG2* expression level, the changes in proliferation was not likely due to apoptosis, rather a change in cell cycle progression. Over expression of *DLG2* resulted in an increased fraction of cells in G2/M phase (Fig. 5f, *p* < 0.001) and the associated decrease in the fraction of cells in G1 phase (Fig. 5f, *p* < 0.001) compared to the mock control, no difference in the number of cells in S phase was detected. Conversely, knockdown of *DLG2* resulted in an increase in the number of cells in G1 phase and a decrease in the number of cells in S phase (Fig. 5f, *p* < 0.001). Over expression and knockdown of *DLG2* was confirmed using qPCR in both SKNAS and NB69 cell lines (Fig. 5g and h). To model the effect of *DLG2* loss, we tested the effect of knockdown of the orthologous *Drosophila dmDLG* gene in a fruit fly model. RNAi-*dmDLG* knockdown resulted in a small decrease in *dmDLG* RNA expression in the larvae (Fig. 5i), the result was confirmed on protein level (Fig. 5j). To confirm if the effect on cell cycle proteins of RNAi-*dmDLG* knockdown was similar to that of *dMYC *over expression (Fig. 3f) we determined the protein expression of Cyclin A and Cyclin B by Western blot. Both Cyclin A and B showed increased expression when compared to the control (Fig. 5j).

## Discussion

To make the case that *DLG2* is the tumor suppressor located in 11q we set forth a criteria based on what is currently known about the clinical and molecular features of deletion of 11q in NB. The first criteria we highlighted were that 11q deleted tumors and *MYCN* amplified tumors are both high-risk tumors that are difficult to treat. This difficultly to treat and resultant poor survival should be evident with altered expression when divided into INSS stage, risk category and overall survival. We could show that low *DLG2* expression is associated with advanced INSS staged tumors (Fig. 1a). Furthermore, we could show that the overall survival and event free survival was decreased with lower expression of *DLG2* (Fig. 1c and d). The second point we raised was that the prospective tumor suppressor gene (TSG) must have an interaction with *MYCN* amplification; either by substituting the function of *MYCN* amplification or by *MYCN* amplification acting to disrupt the 11q TSG function. We have been able to show in multiple datasets with both patient and cell models that *MYCN* amplification results in a decrease in the expression of *DLG2*. Furthermore, we could show in a fruit fly model that the overexpression of the *MYCN* orthologue *dMYC* resulted in decreased expression of *Drosophila dmDLG* and increased expression of Cyclin A and B (Fig. 3g). The effect of *dMYC* over expression on cyclin protein level confirms previous findings [30]**.** After silencing *dmDLG* expression in the fly, we could show that the outcome of the knockdown was similar to that of *dMYC* over expression by altering the same cyclins (Fig. 5j) and consistent with the gene set enrichment analysis (GSEA) that also verified that the genes encoding Cyclin A and B (*CCNA2* and *CCNB1*) significantly correlated to *DLG2* expression in all the analyzed datasets (Table 5). We could also show that induction of *MYCN* in NB cells resulted in a decrease in the expression of *DLG2* (Fig. 3e). Thus, supporting the case that there are common targets of *MYCN* amplification and 11q deletion and that MYCN acts to disrupt the 11q TSG function. It has previously been shown that MYCN has significant function controlling cell cycle and DNA repair in NB [31]. When we examined the gene sequences of *DLG2* and fly *dmDLG* we detected the MYCN/dMYC high affinity binding E-box motif CATGTG [32] appears on multiple occasions within the genes *DLG2* and *dmDLG*. This may provide a mechanism for MYCN to down regulate *DLG2* expression, as MYCN as has previously been shown to do with regulation of *FAIM2* [33], however more investigation into this mechanism is warranted. The third point that we discussed was that there must be a discernable decrease in *DLG2* expression between the 11q-deleted and 11q-normal tumors, and we could indeed observe lower *DLG2* expression in two NB primary datasets (Fig. 4a and b).

We highlighted that the 11q TSG must be involved in the maintenance of DNA integrity as the 11q deleted tumors often show greater chromosomal instability compared to other NB tumors [2]. The *DLG2* GSEA of the NB datasets showed enrichment for Falconi anemia pathway, this is a DNA repair pathway (Tables 1, 2 and 3), the functional loss of which is known to result in an increased number of chromosomal breaks. The mismatch repair pathway, responsible for DNA integrity is also known to be dysregulated in cancers. Furthermore, DNA integrity is one of the key functions of the G2/M checkpoint [34] and is lost if cells rapidly or abruptly leave the S phase. We have shown that *DLG2* both negatively correlates to cell cycle gene expression (Fig. 2a) and regulates the cell cycle, namely by slowing the progression of cells through the G2/M checkpoint (Fig. 5c and f).

As with the other candidate 11q tumor suppressor genes we have tried to detect a suitable second hit or evidence of haploinsufficiency. We have investigated methylation of the CpG island promoter for *DLG2* to determine if there was any discernable differential methylation in the NB samples. We were unable to find any difference in the CpG island, shore or shelf methylation (Fig. 4c and d). We have been able to detect *DLG2* expression for most samples, and furthermore, we have not detected any consistent *DLG2* mutations, suggesting that there is no second hit for *DLG2* but rather a concentration gradient where the lower the expression the worse prognosis. We could show this overall survival and concentration gradient of *DLG2* in Fig. 1f. We could also show that there was no advantage but rather a detriment to the cells when *DLG2* was further knocked down in the 11q LOH cell line SKNAS (Fig. 5a and b). Previous studies have shown that restoration of *DLG2* resulted in an increase in the number of cells paused at the G2-M DNA checkpoint [26], something that we also have shown (Fig. 5c and f). Cyclin B protein level exceeding a certain threshold controls passage through this checkpoint. This is consistent with our fly study finding that Cyclin B is highly up regulated with RNAi-*dmDLG* knockdown (Fig. 5j) and thus allowing DNA damaged cells through the G2-M checkpoint. Furthermore, the GSEA results showed that *DLG2* expression level is inversely correlated to cell cycle and DNA replication genes, further strengthening our functional studies. Interestingly, the tumor suppressor candidates on 11q23 include; *ATM*, *CHEK1* and *H2AFX,* all of which coalesce within the same DNA damage response pathways, which have been shown to be active at the G1 restriction checkpoint. The small amplification of 11q13 has been shown to include Cyclin D, a driving factor pushing the cell into the cell cycle [17]. Therefore, the combination of all of the SROs on 11q can regulate the cell cycle at the different checkpoints and therefore the sum of the alterations may sufficiently dysregulate the cell cycle and account for the haploinsufficiency of all of the candidate genes. These findings are in agreement with the reviewed data by Mlakar et al., for 11q deleted NB as they also reached the conclusion that the 11q deletion is likely going to have haploinsufficiency [11].

## Conclusions

In conclusion, we have provided evidence that gene expression of *DLG2* is disrupted in NB, in particular in the aggressive subsets of tumors. It provides a link between *MYCN* amplified and 11q-deleted NB, as high levels of MYCN down regulates *DLG2* expression, as will loss of one copy of the gene in the 11q-deleted tumors. *DLG2* fulfills the criteria of molecular and clinical characteristics to be an 11q tumor suppressor gene functioning as a G2/M checkpoint.

## Supplementary information

**Additional file 1.**

## Data Availability

The datasets analyzed during the current study are available in the ‘R2: Genomics Analysis and Visualization Platform repository, [http://r2.amc.nl].

## Abbreviations

DLG: Discs large homolog
LOH: Loss of heterozygosity
MYCN: N-myc proto-oncogene protein
NB: Neuroblastoma
SRO: Smallest region of overlap
TSG: Tumor suppressor gene
