# Supplementary material for: Low DLG2 gene expression, a link between 11q-deleted and MYCN-amplified neuroblastoma, causes forced cell cycle progression, and predicts poor patient survival
Source: Cell Commun Signal. 2020 Apr 20;18:65. doi: 10.1186/s12964-020-00553-6 (PMC7171851; doi:10.1186/s12964-020-00553-6)
Supplement: Supplementary file 2 — Additional file 1. [file 12964_2020_553_MOESM2_ESM.pdf]

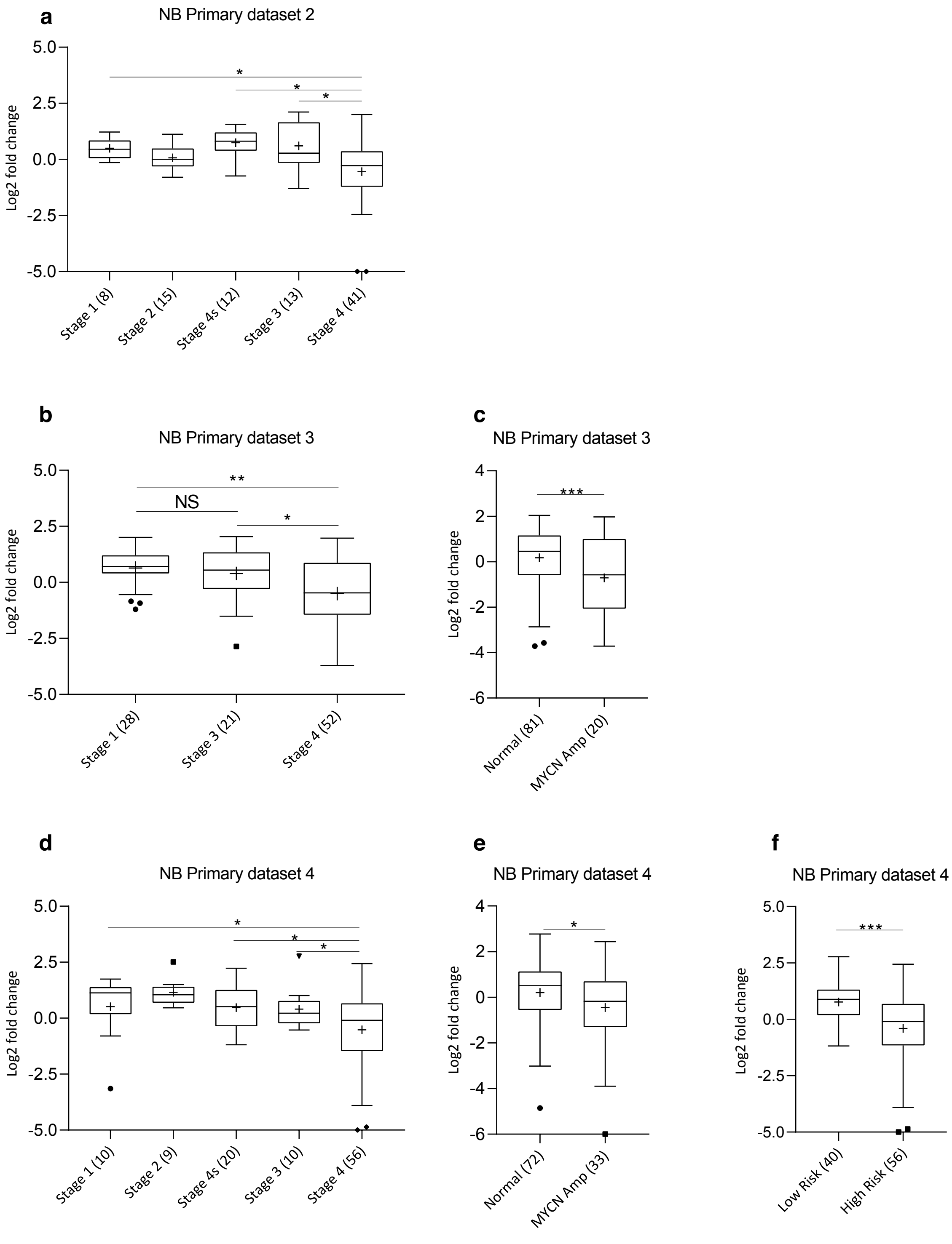

Figure S1. ***DLG2*** **expression in NB correlates with stage, MYCN amplification and Risk**

DLG2 gene expression of primary neuroblastoma datasets 2 (*GSE16476*), 3 (*GSE3960*) and 4 (*GSE73517*) stratified by **a), b)** and **d)** INSS stage, **c)** and **e)** MYCN amplification and **f)** Risk category in primary NB. The expression data are presented as median centered log2 fold change and plotted as Tukeys box and whisker plots showing IQR, line at the median, + at the mean with whiskers ± 1.5-fold of interquartile range. Data outside the whiskers are shown as outliers. $^{*}p < 0.05$, $^{**}p < 0.01$, $^{***}p < 0.001$.

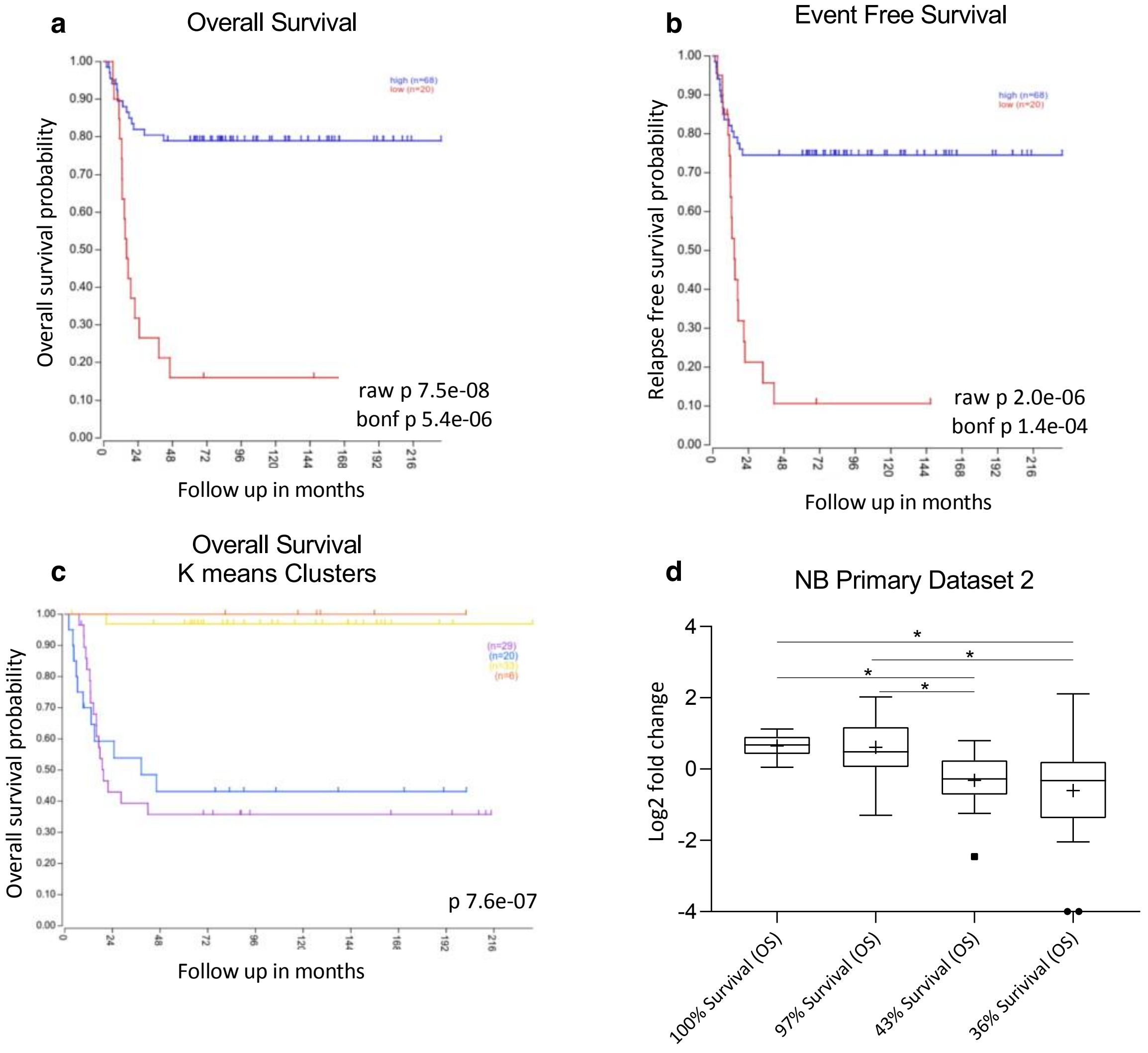

Figure S2. ***DLG2*** **expression in NB correlates with survival**

DLG2 gene expression of neuroblastoma dataset 2 (*GSE16476*), **a), b)** and **c)** are Kaplan-Meier plots showing in **a)** the overall survival probability, **b)** the relapse free survival and **c)** the overall survival of the k means 4 groups clustering. **d)** *DLG2* expression stratified by k means clustering Kaplan-Meier plots. The expression data are presented as median centered log2 fold change and plotted as Tukeys box and whisker plots showing IQR, line at the median, + at the mean with whiskers ± 1.5-fold of interquartile range. Data outside the whiskers are shown as outliers. $^{*}p < 0.05$, $^{**}p < 0.01$, $^{***}p < 0.001$.

Table S1. *Cell cycle, DNA replication, mismatch repair and fanconi anemia pathway genes negatively correlated to DLG2 expression, common to NB datasets 1(GSE49710), 2(GSE16476) and 3(GSE89413)*

**Gene symbol**	**Gene function**	**Cellular process**
ATR	DNA strand break response	DNA repair
BLM	DNA helicase	DNA replication
BRCA1, BRCA2	Double stand break recognition	DNA repair
BUB1, BUB1B	Mitotic checkpoint serine/threonine kinase	Mitotic checkpoint
CCNA2	Cyclin A2, G1/S to G2/M transition	Cell cycle regulation
CCNB1	Cyclin B1, G2/M transition	Cell cycle regulation
CDC6	Cell division, G1/S phase	DNA replication
CDC20	Cell division, anaphase regulation	Chromosome separation
CDC23	Cell Division, G2/M transition	Cell cycle regulation
CDC25B	Cell division, Entry into mitosis	Cell cycle regulation
CDK4	Cyclin dependent kinase, G1/S phase	Cell cycle regulation
DBF4	E2F mediated regulation of DNA replication	DNA replication
E2F1, E2F3	Transcription factor	Cell cycle regulation
ESPL1	Sister chromatid cohesion and separation	Chromosome separation
EXO1	Exonucelease 1	DNA repair
FANCG, FANCI	Cell cycle checkpoint	DNA repair
FEN1	NMEJ pathway	DNA repair
LIG1	DNA ligase, DNA replication and repair	DNA replication
MCM2, MCM3, MCM5, MCM6, MCM7	Initiation of genome replication	DNA replication
MSH6	Mismatch recognition	DNA repair
PCNA	DNA clamp	DNA replication and repair
PRKDC	DNA-dependent protein kinase	DNA repair
POLA1, POLA2	DNA polymerase alpha	DNA replication
POLD2	DNA polymerase delta	DNA replication
POLE2	DNA polymerase epsilon	DNA replication
RAD51, RAD51C	Double strand break repair	DNA repair
REV3L	DNA polymerase zeta	DNA replication
SKP2	S phase associated Protein	Cell cycle regulation
SMC1A	Sister chromatid cohesion and separation	Chromosome separation
RFC3, RFC4	Replication factor, DNA elongation	DNA replication
TP53	Tumor suppressor, DNA binding	Cell cycle regulation
YWHAH	Signal Transduction	Transcription regulation
